# Supplementary material for: Imipramine Accelerates Nonalcoholic Fatty Liver Disease, Renal Impairment, Diabetic Retinopathy, Insulin Resistance, and Urinary Chromium Loss in Obese Mice
Source: Vet Sci. 2021 Sep 9;8(9):189. doi: 10.3390/vetsci8090189 (PMC8473438; doi:10.3390/vetsci8090189)
Supplement: Supplementary file 1 [file vetsci-08-00189-s001.zip › vetsci-1308938-supplementary.pdf]

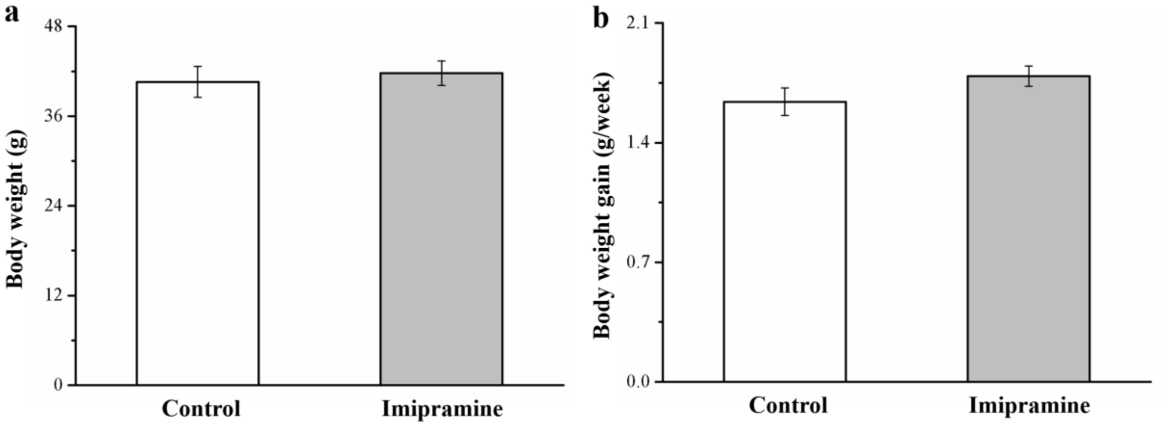

Figure S1. Effect of imipramine (5 mg/kg every day) on (a) body weight and (b) weekly body weight gain in HFD-fed controls and HFD-fed mice treated with imipramine during the 56-day experimental period. No significant differences in body weight and weekly body weight gain were observed between the two groups.

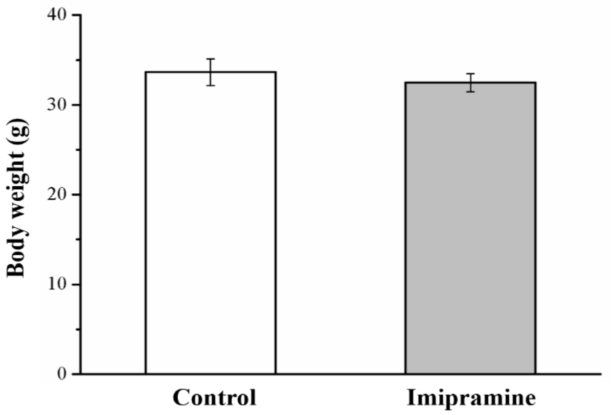

Figure S2. Effect of imipramine (10 mg/kg every day) on body weight in SD-fed controls and SD-fed mice treated with imipramine during the 56-day experimental period. No significant differences in body weight were observed between the two groups.

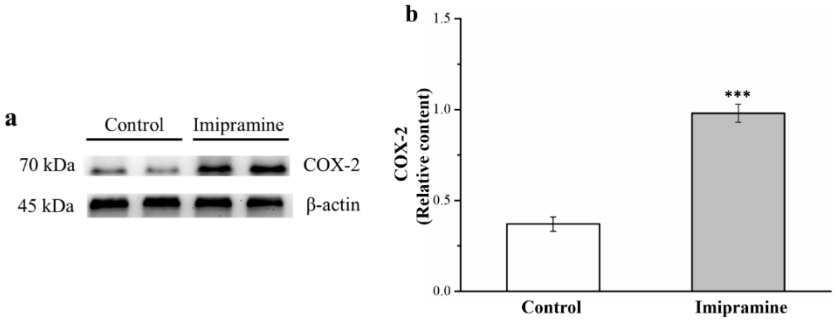

Figure S3. Effects of imipramine (10 mg/kg every day) on (a) a representative image showing blots of liver extracts. The expression level of (b) COX-2 was measured. Data are in the form of mean ± standard deviation (n = 8). ***$p < 0.001$.

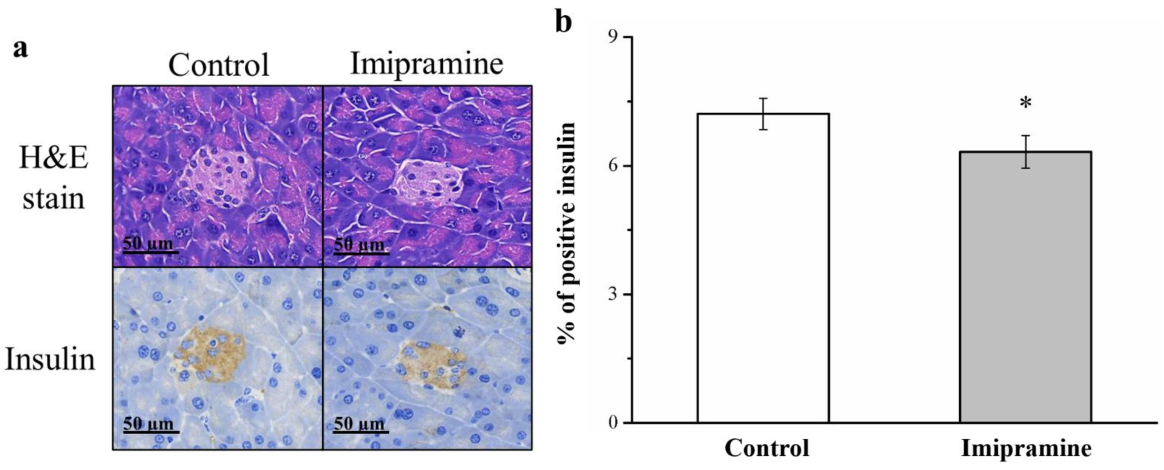

Figure S4. Alterations in (a) islet morphologies (hematoxylin and eosin as well as immunohistochemical stain; 200× magni-fication) and (b) β-cell percentage in control and treatment groups. *$p < 0.05$.

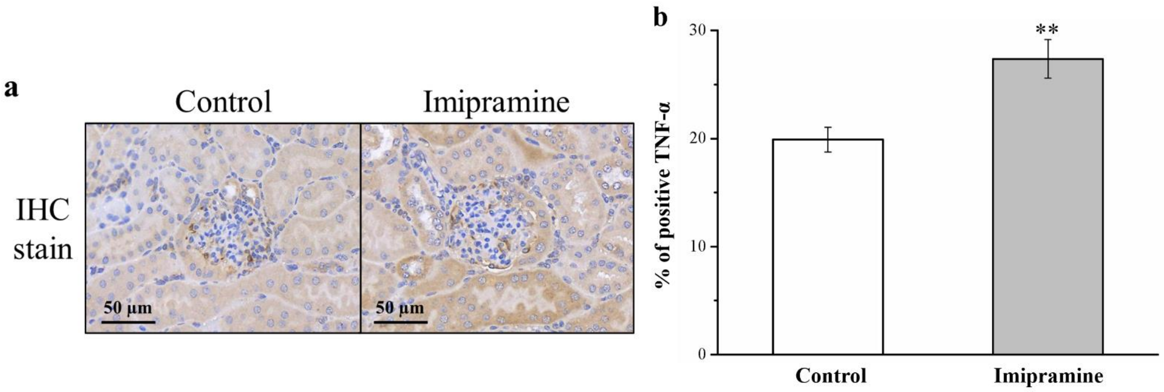

Figure S5. Alterations in (a) IHC stains (magnification: 200×) and (b) renal levels of TNF-α in treatment and control groups. **$p < 0.01$.

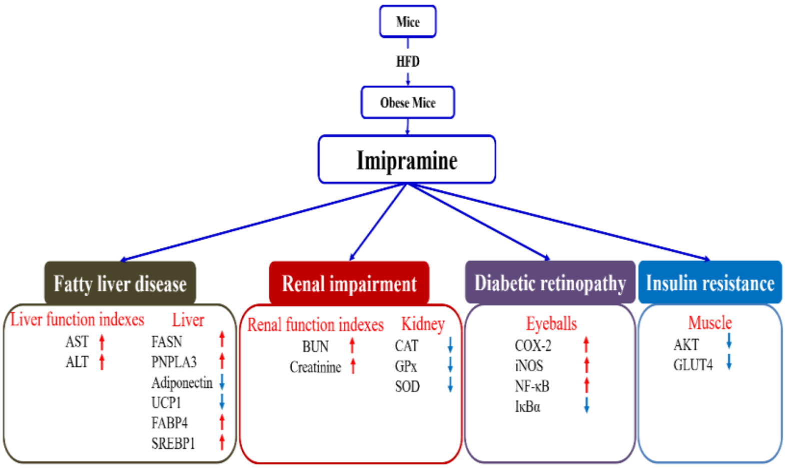

Figure S6. Mechanistic presentation of side effects experienced by HFD-induced obese mice treated with imipramine for 56 days.
